# Biomechanical cues as master regulators of hematopoietic stem cell fate

**DOI:** 10.1007/s00018-021-03882-y

**Published:** 2021-07-07

**Authors:** Honghu Li, Qian Luo, Wei Shan, Shuyang Cai, Ruxiu Tie, Yulin Xu, Yu Lin, Pengxu Qian, He Huang

**Affiliations:** 1 Bone Marrow Transplantation Center, the First Affiliated Hospital, School of Medicine, Zhejiang University, Hangzhou, 310012 Zhejiang People’s Republic of China; 2 Institute of Hematology, Zhejiang University, Hangzhou, 310012 Zhejiang People’s Republic of China; 3 Zhejiang Engineering Laboratory for Stem Cell and Immunotherapy, Hangzhou, 310012 Zhejiang People’s Republic of China; 4 Zhejiang Laboratory for Systems & Precision Medicine, Zhejiang University Medical Center, Hangzhou, 310012 Zhejiang People’s Republic of China; 5 Center of Stem Cell and Regenerative Medicine, School of Medicine, Zhejiang University, Hangzhou, 310012 China; 6 Dr. Li Dak Sum & Yip Yio Chin Center for Stem Cell and Regenerative Medicine, Zhejiang University, Hangzhou, 310012 Zhejiang People’s Republic of China

**Keywords:** Biomechanical factors, HSC, Mechanical forces, Mechanosensor, Cell–cell adhesions, Cilium, Cytoskeleton

## Abstract

Hematopoietic stem cells (HSCs) perceive both soluble signals and biomechanical inputs from their microenvironment and cells themselves. Emerging as critical regulators of the blood program, biomechanical cues such as extracellular matrix stiffness, fluid mechanical stress, confined adhesiveness, and cell-intrinsic forces modulate multiple capacities of HSCs through mechanotransduction. In recent years, research has furthered the scientific community’s perception of mechano-based signaling networks in the regulation of several cellular processes. However, the underlying molecular details of the biomechanical regulatory paradigm in HSCs remain poorly elucidated and researchers are still lacking in the ability to produce bona fide HSCs ex vivo for clinical use. This review presents an overview of the mechanical control of both embryonic and adult HSCs, discusses some recent insights into the mechanisms of mechanosensing and mechanotransduction, and highlights the application of mechanical cues aiming at HSC expansion or differentiation.

## Introduction

**Fig. 1:**
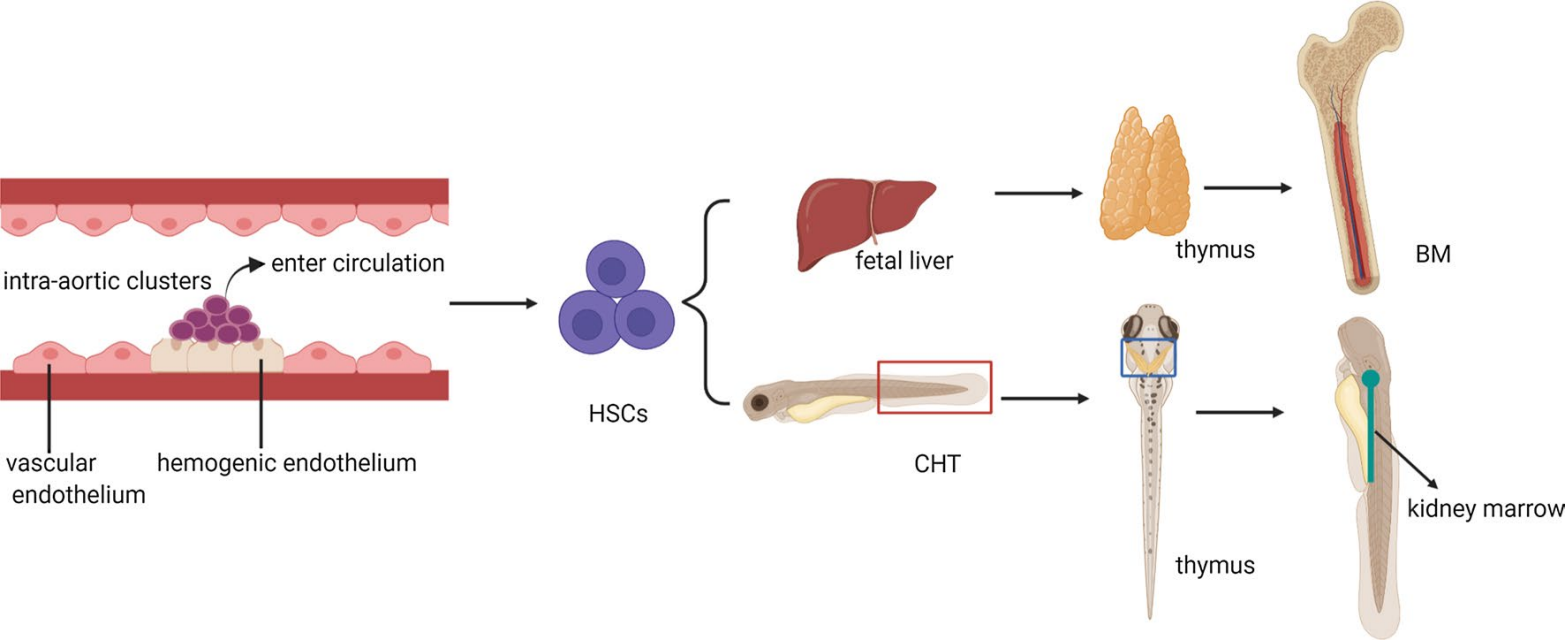
Embryonic development of HSCs in different sites. Intra-aortic clusters giving rise to hematopoietic cells emerge from the ventral wall of the dorsal aorta, from where they enter the circulation, migrate to and colonize in sequential sites including the CHT (red box) or the fetal liver, the bilateral thymus (blue box), and finally the kidney (black arrow) or the BM

Hematopoietic stem cells (HSCs) refer to a very small amount of cell population sitting at the top of the hematopoietic hierarchy. They are responsible for the production of the full complement of blood and immune cells in the body within a unique microenvironment known as niches [1]. Like other stem cells, HSCs also possess the highly complex and controlled ‘SMART’ physiological features of self-renewal, maturation (differentiation), apoptosis, resting mode (quiescence), and trafficking (migration), for maintaining hematopoietic homeostasis in vivo [2]. The origin and maturation of definitive HSCs during development involve a series of successive processes that are robustly templated in time and space with micrometer accuracy [3–5]. In detail, HSCs bud off from the ventral floor of the dorsal aorta (DA) within the aorta–gonad–mesonephros (AGM) region, umbilical, and vitelline arteries via a carefully choreographed and highly conserved process termed endothelial-to-hematopoietic transition (EHT) [6, 7]. Afterwards, they migrate to and develop in sequential anatomical sites of hematopoiesis including the caudal hematopoietic tissue (CHT) in zebrafish or the fetal liver (FL) in mammals, the bilateral thymus, and eventually populate in the kidney or the bone marrow (BM) postnatally. As the dominant site of hematopoiesis in adulthood, BM offers a favorable microenvironment for HSC homeostasis and/or progenitor maturation [5]. A diagram in Fig. 1 illustrates the origin and development of definitive HSCs within different sites during embryogenesis. The nature of the local HSC niche varies along countless spatial and temporal transitions, providing multiple supportive soluble signals and mechanical cues associated with HSC fate decisions and additional fine-tuning of HSC heterogeneity [5, 8–10].

At the molecular level, it is considered that the transcription factor Tal1 plays an indispensable role in acquiring the identity of hemogenic endothelium (HE) whereas Runxl serves as a master regulator of EHT and definitive HSC specification [6, 11–14]. Both in zebrafish and mice, arterial identity is a prerequisite for aortic HE as definitive HE originates from arterial progenitor [15–18]. Many mutants with arterial specification defects (e.g., EfnB2-/- mice) also displayed definitive hematopoietic defects [19], while activation of the arterial program in HE promotes definitive hematopoiesis from human pluripotent stem cell (hPSC) [20]. Then, the subsequent activation of Runx1 mediates the suppression of arterial genes and the upregulation of hematopoietic genes in HE, allowing a further commitment towards the hematopoietic fate. If Runx1 is inactivated, the pre-existing arterial programme in the HE cannot be repressed, resulting in HE maintaining integrated within the DA and failing to undergo the EHT process [15].

The replication of this developmental process will definitely promote the generation of bona fide hematopoietic stem/progenitor cells (HSPCs) with BM reconstitution capabilities in vitro. Therefore, it is of considerable importance to holistically understand the precise mechanisms for instructing the HSC program from both developmentally and clinically relevant perspectives. Researchers were fascinated by the fundamentals of the biochemical regulatory paradigm for decades, and until lately, have paid extensive attention to biomechanical signals due to their equally pivotal impact on the phenotypic specification and functional outputs of HSCs. No single article can be comprehensively concerned with all of the HSC regulatory signaling. This review focuses on the mechanistic principles of HSCs at distinct stages of their ontogeny including embryonic and adult HSCs, predominantly drawing on data from studies on zebrafish, mice and pluripotent stem cells (PSCs), which may provide some insights into future corresponding researches.

## Mechanics in the regulation of endothelium biopsy-beyond the endothelium

Hematopoietic and endothelial lineages have long been considered to be closely related, both of which have actually been demonstrated by quite a few in vitro studies to be derived from a same bipotential mesodermal precursor called hemangioblast. Nevertheless, the presence or lack of hemangioblast in vivo remains fiercely debated and keeps on polarizing the field of hematopoiesis [11, 21]. It has been postulated that the generation of definitive hematopoietic cells in vitro from embryonic stem cells (ESCs) is orchestrated in a stepwise pattern, characterized by discrete developmental stages including the emergence of hemangioblast committed to a DA fate and the formation of a HE intermediate, with EHT being the culmination of these consecutive programming events [11]. Visually, lineage tracing experiments authenticated the endothelial origin of definitive hematopoiesis [6, 22]. Further, human endothelial cells rigorously isolated from distinct hematopoietic tissues including aorta, yolk sac, embryonic liver, and fetal BM all exhibited blood-forming potential when cultured ex vivo [23].

Based on these reasons and more, HE and HSC can be absolutely perceived to be a specialized subpopulation of endothelium and retain endothelial characteristics to some extent. Endothelial cells possess the capability of sensing and discriminating distinct types of mechanical stimuli and responding with unique biological outputs [24, 25]. Therefore, it is tempting to speculate that a number of molecules and proteins involved in endothelial mechanotransduction may also participate in controlling HSC fate. Hence, taking the vascular niche into consideration and appreciating the mechanotransduction process from the angle of endothelium will be of great reference value for the understanding of how mechanical signals regulate HSC biology.

## Mechanics in the regulation of HSC fate determination

HSCs residing in specific niches inevitably perceive a variety of mechanical stimuli such as tensile strain, hydrostatic pressure, fluid shear stress, and even mechanical unloading in microgravity [9]. While subject to mechanical stresses from external loads, cells generate and exert intrinsic forces on the extracellular matrix (ECM) and the neighboring cells reciprocally [26]. Hence, forces generated intracellularly and employed extracellularly are not two entirely separate items but a tensegrity model coexisting and influencing each other, whose interwoven effects control hematopoiesis and maintain the homeostasis of HSCs in an organism [26, 27].

### Intrinsic forces

Intrinsic forces are produced intracellularly in an adenosine triphosphate (ATP)—dependent process by the cross-bridging interactions between actin fibers and myosin filaments [26, 28]. Effects of matrix biophysical features on the fate decisions (viability, morphology, proliferation, lineage commitment) of cultured primary murine Lin-Sca-1 + c-Kit + (LSK) can be selectively eliminated by disrupting the interplay between actomyosin contractility and integrin activation [29], which is in line with an earlier study highlighting the pivotal role of actin contractility in HSC adhesion to extracellular matrices and matrix sensing [30]. Gene expression analysis of human peripheral blood (PB) CD34 + HSPCs using cDNA arrays manifested the expression of several mechanobiological elements such as alpha-actinin, dynein, and dynamin [31–34]. Yet, the precise functions of these genes in HSCs remain to be elucidated.

Additionally, cell-intrinsic forces were tightly associated with cellular function and the biomechanical properties of an individual cell inevitably involved the organization of actin cytoskeletal networks and related regulatory cascades [35]. A label-free microfluidic technique taking differences in cell stiffness as a sorting biomarker can efficiently enrich high-purity live cells because dead cells were generally stiffer than the live ones, whose significant practical superiority was validated by increasing the purity of viable nucleated cells from the samples of thawed cord blood (CB) cells [36]. Compared with mature blood cells, normal BM HSCs appear to be more rigid and less compliant in terms of morphology, accounting for their stable retention within the marrow niche and little mobilization into the circulation [37, 38]. Moreover, cell contractile forces generated by nonmuscle myosin II (NMII), with myosin IIB (NMIIB) being the major one among three mammal isoforms of myosin IIA (NMIIA)-myosin IIC (NMIIC) in human hematopoiesis, contribute to the polarized motility and asymmetric division of adult HSCs underpinning self-renewal and differentiation, which is consistent with earlier reported contributions of NMIIB in the differentiation of megakaryocytes and the asymmetry of erythroid enucleation [39–41]. With functional differences between NMIIA conferring survival and MIIB driving differentiation, the programmatic switch of NMII isoforms from B-and-A to only A occurs, corresponding to the differentiation trajectory of HSCs [39].

The nucleus is a mechanosensitive organelle that is semi-permeable to transcription factors regulated by cytoplasmic biomechanical signaling and, therefore, cellular mechanics is revealed to be highly dependent on the nucleus [42, 43]. Levels of lamins, intermediate filament proteins responsible for the assembly of nuclear structure, seem to control the nuclear tension of hematopoietic cells, which thus results in the discrepancy of trafficking into the bloodstream through the endothelial barrier [37]. Lamins are involved in HSC differentiation as well [44]. Moreover, intrinsic forces can be physically directly propagated to the nucleus through lamin A/C (LMNA), a component of nuclear lamina proteins coupling the linker of nucleoskeleton and cytoskeleton (LINC) complex to chromatin via lamina–chromatin interactions so as to modify chromatin structure and control epigenetic transcription [45]. In leukemia cells, the absence of cytoskeletal mechanical tension and subsequent weak adhesion to BM niches contribute to their chemoresistance and residual disease persistence, prompting leukemia progression and/or relapse [46]. Similarly, in Ptpn21 deletion HSCs, cytoskeleton instability attenuated the quiescence and hematopoietic reconstitution capabilities of HSCs that can be overcome by restoring cellular mechanics [38]. Immediately after, in the following year, the same research team found that the recipient mice inoculated with MLL-AF9-Ptpn21-/-leukemic cells exhibited shortened survival, increased leukemic burden, and more severe leukemic cell infiltration compared with MLL-AF9-Ptpn21 + / + cell recipients. Further data suggested that these phenotypes were independent of the impact on cell signaling but probably a result of cell mechanical alterations (decreased cellular mechanical rigidity and increased cell deformability) in Ptpn21-deleted leukemic cells, strongly implying a biomechanical regulatory role of Ptpn21 in leukemic development and progression [47]. These lines of evidence suggest that in the adult system, cell-intrinsic force plays a vital role in the regulation of HSC morphology, HSC differentiation, and their response to extracellular mechanical stimuli. An important concept arising from recent work of cell mechanics is that long-lived cytoskeletal structures may even act as epigenetic determinants, delivering to and profoundly affecting the behavior of subsequent generations of cells [35]. During embryonic hematopoiesis, it seems that intrinsic force is a likely factor in modulating HSC cell deformation, motility, and migration which underlie their spatio-temporal transitions through distinct anatomical sites. By means of real-time imaging combined with transgenic reporter lines, researchers can clearly visualize the dynamic EHT procedure as morphologically flat HE bends, contracts along with the blood flow orientation until the very end of its departure from the aortic floor, developing into spherical hematopoietic cells [6, 7, 22]. Investigations into the unique biomechanical traits in zebrafish showed that the EHT process is facilitated by the assembly of rings of actin and myosin proteins into anisotropic contractile circumferential actomyosin around stem cells [48]. Poullet et al. observed the morphological alterations of DA endothelium and their collective migration from the sides down towards the aorta floor prior to HSPCs extrusion, compensating surface reduction of emerging HSPCs and hence ensuring overall aorta integrity. Likewise, the actomyosin contractility around the emerging cells drives the final phase of EHT which precisely refers to their individualization from the aorta into the sub-aortic region [49]. Slight cell deformation was also observed in the intravasation of CD41-GFP^low^ multipotent hematopoietic precursors from the AGM into the posterior cardinal vein (PCV), further signifying the existence of intrinsic forces in this dynamic process [50].

Based on above-mentioned findings, it is highly feasible that HSCs can not only interpret changes in mechanical inputs from outside as variations in the presentation of intrinsic forces, but also directly harness intrinsic forces in themselves as tools for manipulating their fate. Nevertheless, the role and mechanism of cell-intrinsic forces in both the adult and embryonic HSCs have not been studied in great detail as of yet.

### Niche geometry

The intricate construction of HSC niches (e.g., BM) serves as a three-dimensional (3D) architectural scaffold and provides a variety of biophysical cues for HSCs and HSC-related accessory cells [26, 51, 52]. To better support and expand HSCs, 3D scaffolds for BM biomimicry have emerged as a preferable approach that fulfills key mechanical requirements of native niches otherwise obscured in conventional 2D culture systems [53–56]. An optimal 3D scaffold for HSC support usually presents the following topographical features, namely adequate surface area for cell attachment, high porosity for cell migration and nutrient delivery as well as alterability in scaffold structure for control of cell interactions [57]. A great amount of research has reported that human umbilical cord blood (UCB) HSCs expand much more robustly in 3D scaffolds than in 2D conditions [58–62]. Murine ESCs (mESCs) exhibited increased survival and proliferation and enhanced differentiation into hematopoietic cells when cultured on electrospun 3D polycaprolactone (PCL) nanofiber in comparison to gelatin-coated tissue culture plates [63].

In BM compartment, HSPCs interact functionally with niche cells which are often referred to as mesenchymal stem cells (MSCs) and/or derivatives thereof [64]. Substrate geometrical features (e.g., nanofiber diameter, pore size and density) have multifaceted effects on these BM niche cells. For instance, human BM-stromal cells (hBMSCs) cultured on a rough surface [arithmetic average roughness (Ra) 11.30 ± 0.43 µm] are more prone to differentiating into osteocytes than those on a smooth surface (Ra 0.05 ± 0.01 µm), with higher secretion of osteogenic-related protein Laminin-5 (Ln-5) and stronger activation of Ln-5 binding integrins [65]. On a microgrooved bearing surface partially mimicking the physiological reticulated microenvironment, mouse BM-derived MSCs showed a twofold to threefold increase in cell proliferation and expressed higher levels of pluripotency-related markers versus a standard 2D culture [66]. Within a certain diameter range (74–148 nm), the ability of TiO2 nanotubes to promote the osteogenic differentiation of MSCs strengthened with the increase of nanosize [67]. Micro/nano hierarchical structures generated by different nanotopographies (nanoneedle, nanosheet, and nanorod) and micropatterns of different sizes (4 µm, 12 µm, and 36 µm) gave rise to significant differences in the osteogenic differentiation potential of hBMSCs and the angiogenesis of human umbilical vein endothelial cells (HUVECs) through macrophage immunomodulation [68]. Substrate fiber orientation, random or aligned, also is a key factor directing stem cell fate [69].

It seems that a similar geometrical machinery conferring cell fate commitment operated in ex vivo cultures of HSPCs as aminated polyethersulfone (PES) nanofiber meshes (a diameter of 529 nm) among PES-based substrates modified by different chemical treatments (amino, hydroxyl, and carboxyl, respectively) exerted the most powerful and positive effect on the adhesion and expansion of human UCB HSPCs [58]. In addition, aminated nanofibers with different spacers, by which amino groups were conjugated to nanofiber surface, also resulted in significant differences in the adhesion and expansion of cryopreserved human UCB HSPCs [59].

As for embryonic niches, in addition to multiple biochemical cues that have been reviewed in other excellent papers [70, 71], physical interactions between HSCs and stromal cells are part of the AGM microenvironment, explant studies confirmed that tissues located ventrally but not dorsally to the DA promotes AGM HSC activity in light of instructive hedgehog signaling from ventral tissues, suggesting that positional information in the AGM compartment plays an important role in the development of functional HSCs and progenitors [72, 73]. Similarly, the abilities of AGM endothelial cells to support HSCs differ based upon their distinct location, as the ventral subregion-derived populations support both HSC maintenance and differentiation while the urogenital subregion-derived populations facilitate HSC maintenance yet fail to induce HSC activity [73–75], implying that cooperative regulation among biochemical signals and physical adhesions is required for embryonic HSC activity.

### Biomechanical properties of the ECM

**Fig. 2:**
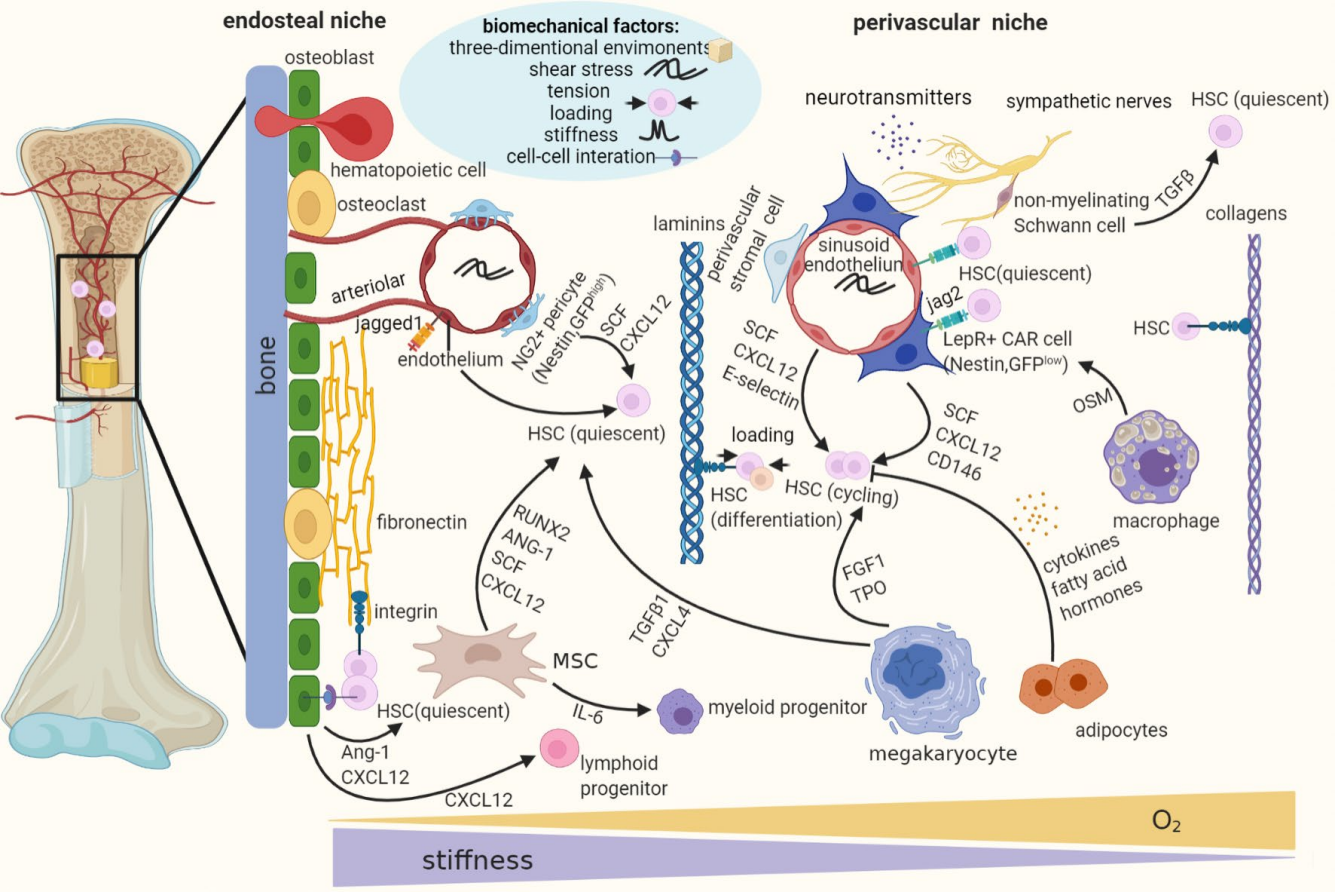
The logistic model of discrete niches in the BM. Adult HSCs are naturally localized in three-dimensional microenvironment of BM which is organized into distinct cellular niches, mainly including the endosteal and the perivascular niches. The endosteal niche, adjacent to the endosteum of the trabecular bone, contributes to maintaining quiescent HSCs while the perivascular (more specifically, arteriolar and sinusoidal) niche, mainly composed of blood vessels and perivascular stromal cells, more activating cell cycle and initiating cell proliferation and differentiation. However, quiescent HSCs associate specifically with small arterioles in the endosteal space, and the most primitive and long-term HSCs are maintained in the perisinusoidal niche [79–81]. Note the presence of biochemical, biophysical and biomechanical factors crosstalking with each other in the in vivo environments. LepR, leptin receptor-expressing; CAR cell, cxcl12-abundant reticular cell; MSC, mesenchymal stromal cell; SCF, stem cell factor; FGF1, fibroblast growth factor 1; TGFβ, transforming growth factor β; NG2, neuron-glial 2; OSM, oncostatin M

Like ECM diversity within other tissues, the structural and physical properties of HSC niches such as stiffness, matrix ligand type, and spatial distribution of adhesive ligands present significant anatomical variations due to their heterogeneities [76]. Taking the BM microenvironment as an example, the endosteum region is replete with abundant fibronectin (FN) and seems to be comparatively stiff (Young’s modulus of 40–50 kPa) while the perivascular space presents a high content of laminin and thus is reported to be softer (3 kPa) [64, 77]. The central medullary region mainly composed of adipocytes and fatty marrow is more compliant (3 kPa) [64]. Analysis of the intact BM of porcine models indicated that the marrow is viscoelastic, with gradations in effective Young’s modulus ranging from 0.25 to 24.7 kPa [78]. The spatial variations of BM niche in cellular and extracellular matrix (ECM) components, soluble, physical, and biomechanical factors are intertwined to create functional niches [1, 79] (Fig. 2).

The cellular morphology of HSCs is closely associated with matrix stiffness because HSCs remain largely round on soft substrates but more scattered on stiffer ones [29]. Moreover, HSCs react to stiffer substrates with increased cell adhesion and motility, which can promote the exit of HSCs from the niche [82]. Colony-forming units (CFUs) assay manifested that more multipotent CFUs (CFU-EM and CFU-GEMM) were generated on stiff (> 44 kPa) relative to soft (3.7 kPa) FN-coated substrates although the effect of matrix ligand cues cannot be totally excluded. It was reflected that matrix ligand type had a selective but significant impact on the lineage specification of HSCs as HSCs cultured on FN-, collagen-, and laminin-coated substrates displayed totally different commitments to myeloid lineage [29]. An analogical inclination was found in ex vivo cultures of mouse BM-derived hematopoietic progenitor cells (HPCs) (LSK) with different matrix stiffness (a shear storage modulus of 50–800 Pa) as Chitteti et al. advocated that higher matrix stiffness facilitated the clonogenicity of LSK cells but lower matrix stiffness seemed to be more related to cell proliferation and differentiation [83]. Moreover, substrate elasticity greatly influences HSPC expansion [30]. Nanopatterns of cell ligands on the matrix also are important physical factors affecting HSC actions as the nanometer-scale spacing between the integrin ligands of the matrix was found to be correlated with HSPC adhesion and subsequent cell signaling transduction [84].

### Biomechanical forces

Adult HSCs residing within the BM or trafficking into peripheral vessels and embryonic HSCs colonizing in discrete anatomical regions at different stages of development are poised to experience biophysical forces [27, 85]. In particular, one of the most important findings concerns the role of blood flow in definitive hematopoiesis [86, 87]. Ever since the initiation of heartbeats and the immediate establishment of blood circulation, vascular endothelial cells, and hematopoietic cells are constantly subjected to hemodynamic forces [87–89]. In general, three types of fluid mechanical forces which are shear stress, circumferential strain as well as hydrostatic pressure are generated because of pulsatile blood flow going through a vessel. Shear stress is the frictional force tangential to ECs while circumferential strain refers to the force perpendicular to the flow direction [90, 91].

With regard to embryonic HSCs, previous studies have unraveled that not only hematopoietic precursors and mechano-responsive vascular endothelium are of developmental and anatomical relevance [92, 93], but also the onset of blood flow and the appearance of hematopoietic cells are temporally connected, suggesting the possibility of blood flow within the “vascular niche” as a local modifier of HSC development [94]. As expected, this original assumption was confirmed in seminal works in zebrafish and mice, and shear stress was the most widely investigated one among the three types of fluid mechanical forces [48, 86, 87, 89, 95–98]. At about the same time, the researches of North TE and Adamo L provided the first experimental evidence that blood flow drives HSC formation [86, 87]. Subsequently, Wang et al. advocated the parallel results in zebrafish [97]. Zebrafish and mouse embryos absent of blood circulation both exhibited a significant reduction in the number of HSCs and severe defects in definitive hematopoiesis [86, 87, 97], one of which, however, can be rescued if exposed to shear stress [86]. Moreover, the application of external wall shear stress (WSS) in ESC cultures and murine embryos induced hematopoietic commitment and enhanced the expansion of hematopoietic progenitors [99]. Most recently, it has been revealed that the impact of the circumferential strain component of blood flow on HSC development is important as well and seems to be conserved between zebrafish and human [95].

Blood flow also contributes to the organization of contractile circumferential actomyosin during EHT and HSPC homing towards the BM [48, 100]. In addition to the luminal hemodynamic forces, DA is constantly exposed to the outer compressive stresses exerted by the surrounding tissues because of embryonic development [101]. The FL is also highly vascularized, yet little is known about the patterns of mechanical forces in this hematopoietic organ for support of HSPC expansion [102].

Adult HSCs sheltered in the BM may not be exposed to blood flow directly. However, BM niche cells do experience fluid flow and could affect HSCs via paracrine signaling. For example, endothelial cells and pericytes might be impacted by the relatively sluggish fluid flow in the blood vessels that feed the medullary cavities of bones throughout the skeleton, further regulating the cycling and quiescence of HSCs [80]. Fluid flow in the lacunar-canalicular network of the bone around osteocytes produces shear stresses of 6–50 dyne/cm^2^, which is implicated in conveying nutrients and signaling elements to osteocytes as well as their mechanical activation [85]. Moreover, it has been reported in mice that a minimal amount of HSCs circulating in the blood flow can experience shear stress that exceeds 600 dyne/cm^2^ in some regions of the aortic walls, a value much higher than the magnitude experienced by cells in human [102]. Mechanical loading, on the other hand, is required for proper BM HSC differentiation. Hematopoietic disorders have been widely reported in humans during exposure to microgravity (spaceflight, for instance), including leukocyte proliferation, reduced number and activity of T-lymphocytes and natural killer (NK) cells, megakaryocyte loss and erythrocyte retention in the BM compartment [103–106], possibly because of the abnormal differentiation potential of BM HSCs under conditions of reduced gravitational mechanical loading [106].

## Mechanosensors and mechanotransduction

On the basis of the above-mentioned research findings, HSCs are somehow endowed with the ability to detect and discriminate a variety of mechanical constituents and convert into different cell actions. However, no consensus is currently reached on how the sensing apparatus and transduction of mechanical signals operates in HSCs. At present, the discovery of numerous promising hints and the proposal of several plausible hypotheses may contribute to unveiling the mystery of how mechanical signals shape the behavior and function of HSCs.

### Cilium

As a hairy-like and microtubule-based (apical membrane) sensory organelle, primary cilia widely exist in mammalian cells such as ECs, embryonic hematopoietic progenitors and almost all human blood and BM cells (97–99%) [107–109]. Cilia have long been considered as the only vestigial evolutionary remnant until being proven by growing evidence to indeed function as a key signaling point decoding miscellaneous mechanical and chemical stimuli in the microenvironment [108, 110]. Notably, calcium channels and receptors are abundant in the ciliary membrane, which further points out that cilia are a communication hub for signal transduction [111, 112]. Human MSCs can sense their mechanical environment through primary cilia, which is required for their osteogenic response and controlled proliferation [113]. Disruption of this mechano-sensory organelle crippled the pro-osteogenic effect of mechanical signals [114]. This is important because both osteoblastic cells and MSCs are essential components of BM niches that support HSCs [115]. Besides, fluid-flow sensing in vascular endothelial cells depending on primary cilia was shown to regulate the biosynthesis of nitric oxide (NO) [116, 117], which is a well-known essential modulator of HSC functions [118]. As BM HSCs are often found in close proximity of the vasculature [119], whether mechanical stimulation of NO production through primary cilia is sufficient to further influence the outputs of HSCs remains unclear but is an intriguing possible mechanism for regulation of HSC fate.

As for developmental HSPCs, a canonical “9 + 0” axoneme 3D ultrastructure of primary cilia was directly visualized in zebrafish vascular endothelium in the AGM region, and a primary cilia-dependent Notch signaling axis was found to be required for HE specification. Primary cilia-dysfunction embryos exhibited severe HSPC defects that can be prevented by the overexpression of the Notch intracellular domain (NICD) [108]. Furthermore, Notch target gene (ephrinB2a), on the other hand, was downregulated in blood flow-deficient embryos [87, 97], in combination with previous evidence showing that endothelial primary cilia can mediate blood flow in the zebrafish vascular development [107] and above-mentioned studies demonstrating that blood flow is a positive regulator of HSPC development, it is reasonable to hypothesize that primary cilia may act as a mechanotransducer relaying signals from blood flow to embryonic HSPCs. These events strongly suggested the affirmative role of cilia in mechanosensing and mechanotransduction in spite of remaining to be definitively demonstrated in HSCs.

### Nitric oxide

As a diffusible gas transmitter generated from arginine by nitric oxide (NO) synthases (NOS), NO exerts a broad effect on cellular biological activities including mechanotransduction [87]. When encountering blood flow, vascular endothelial cells are capable of producing NO, functioning as an essential vasodilator in the regulation of vascular tone [117]. Both fluid shear stress and vertical mechanical stretch can trigger the rapid production of NO [120–122]. Three NOS genes were found in the mammalian genome, referring to the neuronal, endothelial, and inducible NOS isoforms (nNOS, eNOS, and iNOS, respectively), each of which can be reliably detected in mouse BM [118]. Aleksinskaya et al. provided the first experimental confirmation of free NO radicals in rodent BM using a NO spin trapping and electron paramagnetic resonance spectroscopy. Besides, eNOS is the dominant source of basal NO (66%) and the iNOS isoform also accounts for a significant proportion (23%) [123]. Moreover, various biological steps in adult HSCs/HSPCs are regulated by NO signaling and the modulation depends on HSC source because opposite effects can be observed when HSCs of different origin are compared. For example, it induced BM HSC proliferation and myeloid differentiation and reduced their capacity of long-term reconstitution [124] but promoted the homing and engraftment of CB HSC [125]. Therefore, the exploitation of NO-releasing agents or the pharmacological activation of NO-dependent intracellular pathways to bolster the number or activity of HSPCs are suggested to be a promising strategy suitable for therapeutic applications [125]. However, another study demonstrated that iNOS-deficient mice were easily mobilized and their BM-derived mononuclear cells were endowed with intensified homing and engraftment [126]. NO depletion in human PB HSCs led to their shift from differentiation to proliferation [127].

Physiological functions involving NO have also been described in developing HSCs. As a process resembling embryonic HSC budding, NO-induced endothelial podokinesis plays a permissive role in mediating the execution of the vascular endothelial growth factor (VEGF)-guided program of directional endothelial cell movement by interfering cell-ECM adhesive interactions [128]. With regard to definitive hematopoiesis, the researchers hypothesized that NO produced locally by endothelial cells must also affect HSC formation since DA is the requisite de novo site for HSC emergence [129, 130]. As expected, a wide spectrum of independent studies has successively corroborated that NO signaling is required in vascular niche for blood flow-dependent HSC generation during embryogenesis. Ectopic NO strengthens hematopoiesis and mitigates hematopoietic defects resulting from cardiac dysfunction, while abrogating NO signaling would diminish the pro-hematopoietic effects of blood flow [86, 87, 97]. Parallel results were reported in phospholipase C gamma 1 mutants (*plcg1*-/-) that the perturbations of arterial specification and HSPC formation caused by the absence of blood circulation can be prevented by Ginger-induced robust NO upregulation [131]. In zebrafish embryos, NO synthase (nNOS and iNOS) appears to be directly activated by klf2a, which is required for blood flow-dependent HSC maintenance [97]. Further, NO was found to lie downstream of Runx1 based on the ability of NO antagonists in significantly attenuating the expansion of hematopoietic progenitors induced by shear stress without affecting the upregulation of Runx1 [86].

To sum up, in embryonic HSCs/HSPCs, NO often serves as a definite downstream player of blood flow that mediates its positive effect on HSPC formation. Its impact on adult HSCs/HSPCs, however, is largely obscure due to quite contradictory conclusion among different literature, which we think might be a result of distinct analyzed HSC types. As NO is a locally acting signaling molecule and HSCs of different origin (BM HSCs, CB HSCs, and PB HSCs) occupy totally different niches.

### Mechanically gated ion channel

Plenty of flow-modifying agents that regulate the formation of AGM HSCs are indeed well-known modulators of ion channels. Thereinto, Ca2 + -channel blocker nifedipine and Na + /K + fluxes modulator glycoside digoxin both enhance HSC formation, while BayK8644, a potent Ca2 + -channel activator, diminishes the number of HSCs [87]. As a major class of ion channels, cationic stretch-activated channels (SACs) are capable of sensing mechanical forces with high sensitivity and wide dynamic range and permeable to calcium (Ca2 +), a significant second messenger implicated in cell fate decisions [132, 133]. Thereinto, piezo channels can be triggered by the NMII-dependent intracellular traction forces in response to mechanics like substrate elasticity and matrix topography, governing the mechanosensitive fate of stem cells [134, 135].

Calcium flux has been documented in the AGM-derived cells exposed to WSS, stimulating intracellular calcium signaling that directly potentiates the production of prostaglandin E2 (PGE2) responsible for hematopoietic potential modulation, in agreement with the identification of calcium signaling as the second most enriched pathway in WSS vs. static cultures of AGM-derived cells [89]. The deformation of primary cilia like bending induced by mechanical stimuli causes intracellular Ca2 + oscillation followed by modifications in calcium signaling cascades [110]. Moreover, it is well appreciated that eNOS binds calmodulin, whose activity is regulated by Ca2 + [136]. Many events sensitizing eNOS to Ca2 + can stimulate the release of NO [137].

### Crosstalk with known signaling pathways controlling HSC development

In addition to unique mechanosensation and mechanotransduction, the activation of multifarious well-established developmental HSC regulatory signaling pathways was identified in endothelial cells exposed to force, suggesting their potential interplay within HE and/or HSCs [138, 139].These classic regulatory transcriptional programs include PGE2, Wnt, Hedgehog, Notch, bone morphogenetic protein (BMP), and VEGF signaling. Expectedly, ingenuity pathway analysis showed that Prostaglandin, Wnt (especially non-canonical Wnt) and Notch signaling all manifest varying degrees of upregulation after being exposed to WSS and elevated PGE2 production is obvious in the AGM mediated by calcium flux [89]. PGE2 production can be induced by WSS to control the expansion of hematopoietic populations in the developing embryo. BMP was identified as a downstream target of shear stress-protein kinase A (PKA)-cAMP response element-binding protein (CREB) pathway for promoting HSC emergence [98]. The rescue of arteriogenesis and hematopoiesis by ginger treatment in aforementioned VEGF pathway mutants plcg1-/- displaying complete disruption of blood flow are BMP and Notch-dependent [131]. Shear stress promotes the formation of a mechanical sensor complex composed of VEGF receptor 2 (VEGFR2 also called FLK1), vascular endothelial (VE)-cadherin and β-catenin [140]. These wide-ranging possibilities of complicated crosstalk under mechanical conditions provide a cooperative control system working in concert to dictate HSC fate.

### Cell–cell and cell–ECM adhesions

Cells are not just as a single entity with innate cytoarchitecture and cytoskeleton, but also in the condition of concatenating adjacent cells and the ECM via cell–cell and cell–ECM adhesions [26]. Likewise, both in embryonic and adult HSCs, interactions between HSCs and ECM/supportive cells are essential microenvironmental constituents of various HSC niches closely relevant to HSC fate [74, 141–143]. Here, we will emphasize some key adhesive molecules or structures that are known to play a crucial role in HSCs and/or the mechanotransduction process.

During development, the engagement of Notch signaling in HE specification requires posterior lateral plate mesoderm (PLPM) cells migrating over the somite boundary and their close physical contact with somatic cells via ITGB1-mediated adhesion to FN [144, 145]. Within FL hematopoietic compartment, some HSPCs were observed in both the luminal and parenchymal aspects of sinusoidal endothelial cells ECM comprised of laminin and FN, interacting with sinusoidal endothelial cells through endothelial protein C receptor (EPCR) [146]. Time-lapse live imaging of HSPCs in the zebrafish embryo also revealed striking physical anchorage of HSPCs to perivascular endothelial cells in the CHT niche microenvironment which further orient their mitotic divisions [143]. Imaging of the mouse BM uncovered that most quiescent HSCs are adjacent to arterioles [146]. Malignant HPCs like leukemia blasts often exhibit aberrant adhesive structures and signaling and that targeting adhesion signaling is considered as a potential strategy of rational anti-leukemia therapy [147–149].

These junctions are often sites for mechanical convergence capable of sensing and conveying physical forces, of which cadherin-mediated cell–cell adhesions and focal adhesions (FAs) are the most remarkable ones [150, 151]. Specially, VE-cadherin expressing cells stands for a primitive HSC population [152, 153]. Besides, EHT was regarded as a partial epithelial to mesenchymal transition (EMT) process featured by a transitional phenotype of post-EHT cells situated in the intra-aortic clusters with decreased VE-cadherin-mediated endothelial cell–cell and MMP2-mediated cell-ECM interactions [154, 155] Biomechanical forces derived from blood flow have been reported to promote the dissociation of the post-EHT HSPC clusters into individual HSPCs [87]. Previous findings highlighted the junctional stability of cadherins to withstand mechanical forces and their mechanosensitive ability for variations in substrate rigidity [153, 156, 157]. In VE-cadherin-null endothelial cells, several flow-responsive actions were absent [140]. As another mechano-responsive receptor localized to cell–cell junctions in endothelial cells, platelet endothelial cell adhesion molecule-1 (PECAM-1) became activated under fluid shear stress, transmitted mechanical signals to VE-cadherin and activated VEGFR2 [158]. CD44-mediated cell rolling interactions underpinning lymphocyte trafficking and HPC homing were strengthened by tensile mechanical force through inducing the rapid allosteric transition of CD44 to a high-affinity state [159]. Similarly, HSPC homing was directed by the rolling adhesion of HSPCs to endothelium via selectins upon shear stress. Adhesion reorganization within HSPCs was observed during this process, corroborating previous studies demonstrating that mechanical stimuli play a positive role in regulating the composition and kinetics of adhesive junctions [100, 160–162].

Cell-ECM interactions enable cells to sense and react autonomically to the mechanical cues of their context. Also present in HSPCs, cell-ECM contacts are mechanical sensors and/or mechanotransducers of matrix elasticity [30]. The intracellular transmission of mechanical stimuli like topography and forces is attributed to the effect of cell-ECM attachment. In brief, mechanical stimuli activate integrins at cell-ECM adhesion and intrinsic forces are utilized by cells to form mature FAs, a common load-bearing anchorage site, acting as mechanosensitive rheostats to drive single-cell mechanical homeostasis [163–165]. Postulated mechanisms of mechanotransduction include but are not limited to the specific type of integrin receptors expressed in cells and ligands present in the ECM, given the nonspecific characteristics of mechanical inputs [26, 159]. As described for anchorage—dependent cells, it has also been revealed for HSCs that multiple ECM proteins such as FN, laminin and collagen provide structural form and modulate their behavior possibly through integrins [64, 166–168], because it is well-established that these ECM proteins shared common integrin recognition motif termed RGD (Arg–Gly–Asp) [85]. Also, many substrates do actually elicit mechanosensitive responses in HSCs through integrins [39, 82, 169], which may be a likely mechanism for the regulation of HSCs by distinct matrix stiffness. Notably, integrin aIIb (CD41) featuring low-level expression in developmental emerging HSCs in the AGM region is specifically used as a nascent HSC marker [170, 171]. Within FL hematopoietic niche, the interaction between HSPCs and ECM is dependent on β 1-integrin present on the HSPCs binding to vitronectin and FN generated by hepatoblasts [142].

In addition, the recruitment of FA kinase (FAK) to FAs is required to relay external mechanical information into cells. The ratio of phosphorylated FAK to total FAK which symbolizes the activity of FAK dramatically increased with substrate stiffness. Blocking of FAK activity in myoblasts led to abrogated stretch-induced alignment and differentiation [172, 173]. FAK plays a predominant role in the activation of the Rho family of small GTPases (Rho-GTPases) [174] which are mechanotransducers responsible for relaying signals from blood flow to YAP in the embryonic HSPC production [95]. Besides, cell-ECM interactions have a bilateral effect on mechanotransduction, while matrix stiffness or loading influences the behavior of cells which in turn exert traction forces on the ECM and secrete ECM remodeling proteins like matrix components or proteases to strengthen or degrade the ECM and enhance or cleave adhesive interactions [175, 176]. The HSC niche is particularly dependent upon ECM remodeling proteins to control HSC quiescence and mobilization and hematopoiesis [154, 177, 178]. All in all, although the role of cell–cell and cell–ECM interactions in HSCs has been studied extensively, a detailed picture of adhesion force transduction is still lacking.

### Mechano-responsive transcription factors

Mechanical effects can be converted into protein-level through mechano-responsive transcription factors (TFs). Yes-associated protein (YAP) and Transcriptional coactivator with PDZ-binding motif (TAZ) are two well-appreciated mechanics-induced transcriptional coactivators [179]. In most cases, YAP/TAZ activity is restricted to cells experiencing mechanical stresses [180]. YAP activation can be triggered by different types of mechanical cues such as substrate stiffness, cyclic stretch, and shear stress through facilitating its sub-cellular translocalization from the cytoplasm to the nucleus [95, 179, 181]. YAP is also required for the mechano-morphogenetic process by controlling actomyosin-mediated tissue tension [182, 183]. Goode D. K and his colleagues observed a precise temporal nuclear localization of YAP in endothelium just before the EHT process during murine hematopoietic development. Further functional validation experiments identified that TEAD/YAP interaction is a stage-specific regulator necessary for early hematopoietic specification [184]. Most recently, it has been shown that YAP activation and the up-regulation of YAP target genes are sensitive to the cyclic stretch and inform HE commitment towards HSPC fate, for the first time confirming a connection between biomechanical cues and YAP in determining HSC fate [95].

KLF2 serves as another crucial mechano-activated TF whose expression mirrors the onset of fluid shear forces in the developing mouse embryos [185], and is an immediate responder to shear stress in mouse ESC-derived CD41 + Flk-1 + cells and during ESC differentiation towards hematopoietic and endothelial potential [86, 99]. Likewise, it was found that the expression of its zebrafish ortholog, klf2a is dramatically reduced or even absent in the vasculature without blood circulation. In addition, klf2a is a pivotal mediator in blood flow-induced HSC production [97].

As a member of the basic leucine zipper (bZIP) TF family, cAMP response element-binding protein (CREB) can also be activated by miscellaneous mechanical loadings [186–188] and trigger the endothelial and hematopoietic differentiation of ESCs via their recruitment to the Etv2 promoter [189]. Moreover, it has lately been described to be abundantly expressed in the AGM and act as a downstream effector of fluid shear stress affecting the EHT process and HSC emergence through a PKA-CREB-BMP signaling pathway [98]. Roughly in agreement with this experimental data, Ncx1 heartbeat mutants and static cultures of AGM exhibited a significant reduction in the phosphorylation of CREB and prostaglandin E2 (PGE2)-cAMP-PKA signaling axis mediated the effects of WSS on definitive HSCs [89].

### Cytoskeleton

As a cellular interconnected scaffold composed of three main polymers including actin filaments, intermediate filaments and microtubules, cytoskeleton forms intricate structural networks with different architectures [35]. It is reported that the primary machinery of cellular force-sensing and force-generating comes from changes in the cytoskeleton [35, 190, 191]. Cell-intrinsic forces are generated through either the concerted polymerization or slide of actin filaments along the bipolar filaments of NMII [190, 192]. The suppression of NMII activities attenuated the expansion of LSK cells conferred by matrix elasticity [30]. Moreover, the extent of cytoskeletal contractility was proportional to the degree of adhesion strength [193, 194]. Therefore, cytoskeleton has important implications for mechanical transmission as a hub of communication between cells and the external physical microenvironment [179, 180, 195]. When external mechanical stimuli were directly exerting on the cytoskeleton or on the transmembrane adhesive receptors that were connected to the cytoskeleton, cytoskeleton remodeling and cytoskeletal tension rearrangement occurred under the action of a myriad of actin-binding proteins [196, 197]. Endothelium bearing steady laminar shear stress exhibited a typical phenotype of “apical stress fibers” enriching robust actin- and myosin-containing filaments in the apical cell membrane because of cytoskeleton-related gene activation [24]. Similar “apical constriction” event also occurred in EHT cells which may rely on the activity of actomyosin recruited at the circumferential actin belt controlled by myosin regulatory light chain 9 (Myl9) [48].

Apart from focusing on the structural changes of cytoskeleton, researchers have long observed the upregulation of cytoskeletal proteins induced by forces [198]. In addition, cytoskeleton was identified as a key regulatory input for YAP/TAZ due to the abrogation of YAP/TAZ activity by Cofilin, CapZ, Gelsolin, and other F-actin-capping/severing proteins and the enhancement of YAP nuclear translocation by cortical actin bundling in response to shear stress [180, 181]. Another research on mouse kidney development also revealed that CDC42 Rho-GTPase, a well-established promoter of F-actin polymerization, contributes to the nuclear retention of YAP [199]. Rho-GTPase mediated blood flow-induced YAP activation and HSPC production in zebrafish embryos and in vitro [95]. Defective cytoskeletal architecture in HE or EHT undergoing cells followed by the abrogation of blood flow resulted in the severe impairment of their morphodynamics and thus higher susceptibility to cell death, which may explain a significant decrease in the number of HSPCs previously reported upon blood flow obliteration treatment [4, 48, 200].

**Fig. 3:**
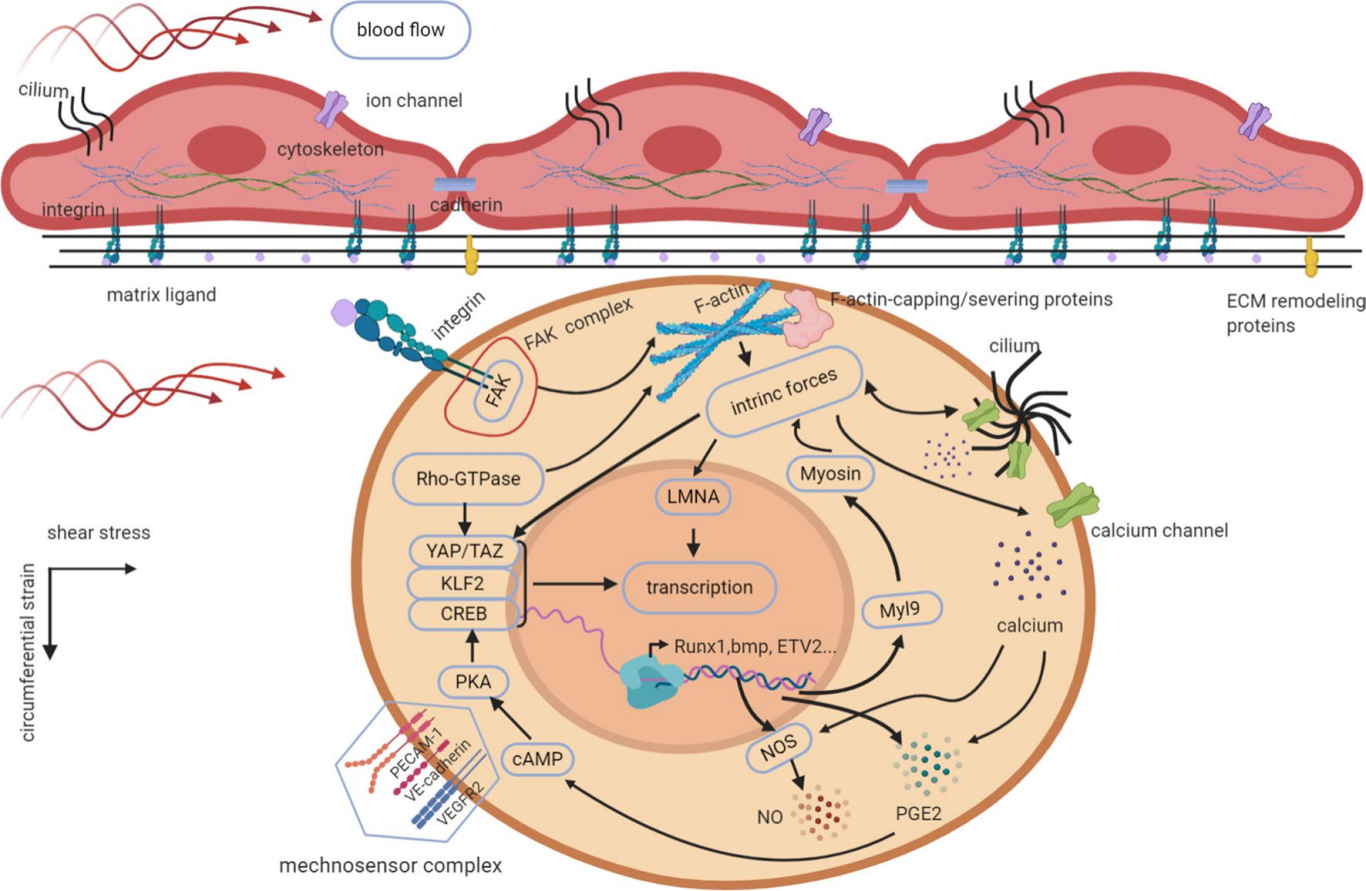
Schematic representation of molecular mechanisms translating biomechanical cues into the cellular genetic program in HSCs. Biomechanical inputs from external loads directly stimulate mechanosensors such as mechanically gated ion channels, adhesion receptor-ligand bonds, cytoskeleton and primary cilia. PECAM-1, VE-cadherin and VEGFR2 constitute a mechanosensor complex together. Intrinsic forces are generated under environmental mechanical constraints, transmit to neighboring cells through junctional interfaces, and consequently elicit cellular mechanoresponses. For example, blood flow governs the heterogenous organization of circumferential actomyosin and actin/junction contacts characterizing HSC emergence via a Myl9-dependent mechanism. Besides, intrinsic forces can directly pass on to the nucleus through LMNA, affecting chromatin structure and thereby controlling epigenetic processes. Biomechanical cues cooperate with biochemical signals. For instance, the expression and/or activation of mechanics-responsive TFs including YAP, KLF2, and CREB induced by fluid forces drives HSC programming. Interactions between these mechanical molecules are common, one of which is that PGE2 and NO production have been revealed as the downstream events of calcium flux triggered by mechanical stress that involved in hematopoietic regulation

Below, we mainly highlight the key molecular mechanisms involved in the regulation of HSC actions under mechanical conditions, illustrated in Fig. 3.

## In-vitro mechanical mimicry platforms for HSC support

Research aiming to recapitulate the developmental processes of HSCs in vitro relies heavily on the establishment of robust and reproducible methods, one of which is the effective biochemical and biomechanical simulation of in vivo milieus. However, common HSC supportive strategies are confined to biochemical exposure like the delivery of various growth factors and cytokines, with only meager attention paid to the effects of biomechanical cues. Systems of this kind enhance HSPC proliferation but induce substantial differentiation [201, 202]. To circumvent this limitation, mechanical engineering and materials science where biomechanical cues such as substrate elasticity/rigidity, micropattern, nanotopography, and externally applied forces are well-designed have thrived to be more conducive to achieving sustained expansion of HSCs while keeping their stemness and multipotency [56, 203]. Among them, specially fabricated 3D nanofibers were most frequently reported in the literature [56, 203–205], which are often hierarchically structured scaffolds constituted by different biomaterials including natural polymers such as fibrin, collagen [205], tropoelastin [30] and silk fibroin [60], synthetic polymers such as PCL (an extensively used scaffold material  in tissue engineering), hydroxyapatite (HA, one dominant component of bone), polyethylene terephthalate (PET), PES, hydrogel, polyurethane (PU), and poly L-Lactic (PLLA), ceramics and a hybrid of them. Electrospun nanofiber ceramics have been lately reported as bone mimicking materials [206]. Selected studies carried out in the last 5 years on the expansion and differentiation of HSCs utilizing synthetic nanofibers are compiled and highlighted in Tables 1 and 2. All these 3D nanofiber platforms have been adopted as analogs of the BM niche. By altering the ratio of distinct constituents, crosslinking levels, concentrations, bond with other components, and the density of cell adhesion ligands, biomaterials are mechanically tunable to generate nanofibers equivalent to those of the natural ECM (in size, structure and elasticity) [207].

**Table 1:** State-of-the-art nanofiber technology-based niche models for HSC expansion

Author and year	Cell source	Culture duration /initiation of cell seeding	Nanofibers	Medium and cytokine cocktail	Culture type	TNC	CD34+/CD133+	CD34+/CD133+					Hematopoietic engraftment capacity	Reference
								CFU-GEMM	CFU-GM	BFU-E		Total		
Mousavi et al. (2018)	CB-CD34 +	10 days/10,000	PCL	Stem line II	Static 2D culture (TCPS)	38.3-fold	5.75%	–	–	–		–	–	[203]
				TPO/SCF/FLt3	3D culture (PCL + FN)	58-fold	65.65%	2.1	1.1	1.5		1.3		
Braham et al. (2019)	CB-CD34 +	10 days/250,000	Hydrogels (bioactive matrigel/bioinert alginate)	IMDM TPO/SCF/FLt3/IL-3/IL-6	Hydrogels + no feeder (negative control)	–	–	–				–	–	[211]
					Hydrogels + MS-5 (positive control)		–	–						
					Hydrogels + MSC		–	–						
					Hydrogels + EPC		–	–						
					Hydrogels + A-MSC		–	–						
					Hydrogels + O-MSC		–	–						
					Hydrogels + MSC + EPC		–	–						
					Hydrogels + A-MSC + EPC		–	–						
					Hydrogels + O-MSC + EPC		–	–						
					Hydrogels + A-MSCs + O-MSCs + EPC		Matrigel maintained CD34 + CD38- and increased CD34 + CD38 + HSPCs; Alginate more enhanced CD34-CD38 + cells	Maintain CFU-GEMM Progenitors						
Bai et al. (2019)	CB & BM-CD34 +	24 days/(2–4) × 10^6^	Zwitterionic poly- based hydrogels (ZTG)	StemSpan SFEM II	TCPS flasks	1450-fold	8.4%(22-fold)	–					–	[212]
				SCF/FLt3/TPO/IL-3/IL-6/FL	Optimized Delta1^*ext*−*IgG*^ culture	530-fold	34.9%(122-fold)	–					–	
					Optimized ZTG culture	322-fold (TCC)	93.7%(319-fold)	No significant difference with uncultured groups (BM)					Largest increase of LT-HSC, highest levels of engraftment and multi-lineage repopulating capabilities	
Kang et al. (2016)	CB-CD34 +	7 days/50,000	PCL + HA	CellGro	TCP	35.1-fold	5.20%	–					–	[213]
				FLt3/TPO/SCF/IL-3	PCL	141.eightfold	32.20%	CFU-G & CFU-M predominated						
					PCL + collagen type I	146.fourfold	29.87%	BFU-E predominated						
					PCL + FN	163.eightfold	31.80%	Various colony types						
Pan et al. (2017)	CB-CD34 +	14 days/10,000	PCL	IMDM	2D culture CD34 + -hAMSCs	111 × 10^4^	3.2%(3.6 × 10^4^)	–					–	[214]
Islami et al. (2017)	CB-CD133 +	7 days/50,000	PLLA	Stem span FLt3/TPO/SCF	2D culture	–	35.60%	0.48	0.68		–	0.5		[215]
					Unmodified PLLA		57.40%	1	2.5		–	1.25		
					PLLA-Collagen		74.70%	1	2.72		–	1.35		
					PLLA-FN		85%	1.16	2.88		–	1.48		
Arabkari et al. (2019)	CB-CD34 +	7 days/250,000	PES	Stem span FLt3/TPO/SCF	2D culture (liquid culture)	10.51-fold	75.19%(4.86-fold)	1.9	2		2.2	2	–	[62]
					3D culture (aminated PES)	16.08-fold	53.88%(9.17-fold)	2.7	2.7		3.1	2.8		
Darvish et al. (2019)	CB-CD133 +	7 days/10,000	PLLA	Stem span FLt3/TPO/SCF	2D culture		35.60%						–	[216]
					2D + MSC		45.60%							
					PLLA(3D culture)		57.40%							
					PLLA + MSC		88.70%							
Eskandari et al.(2015)	CB-CD133 +	14 days/50,000	PES	Stem span	2D culture	–	24.40%	–				–		[217]
				FLt3L/TPO/SCF	PES-FN (3D culture)		36.90%	Higher total colony counts compared with 2D						
Batnyam et al. (2015)	CB-CD34 +	7 days/2500/ml	PU	StemPro-34 FLt3/TPO/SCF	2D culture									[218]
					3D culture I (transversely isotropic)	1.8 × 10^5^/cm^2^	11,000/cm^2^	2.6	3.9		3.7	–		
					3D culture II (anisotropic)	3.3 × 10^5^/cm^2^	27,000/cm^2^	6.2	8.5		8.6			
						3.5 × 10^5^/cm^2^	25,000/cm^2^	9.7	10		11

**Table 2:** State-of-the-art nanofiber technology-based niche models for HSC differentiation

Author and year	Cell source	Nanofibers	Initiation of cell density	Medium and growth factors	Approach	Hematopoietic related markers	CFU potential	Hematopoietic engraftment capacity	References
Dehdilani et al. (2016)	Mouse-ESCs	PCL	7 days/2500 cells/100_l	IMDM + LIF	PCL	72.08%/52.27%	–	–	[63]
				SCF/IL-3/IL-6/FL	2D culture (gelatin-coated TCP)	55.92%/43.51%			
						(CD34 +)			
Xu et al.(2016)	Human-iPSCs	Hydrogel	10 days/(1–2)*_*106 cells/ml	StemSpan^TM^ SFEM II	Hydrogel	78.80%	–	–	[56]
				BMP4/SCF/FLt3L/VEGF/	Hydrogel + BM-stromal cells + factors	71.60%	–	–	
				PGE2/IL-3/IL-6/GM-CSF/G-CSF/EPO	Hydrogel + BM-stromal cells + factors	47.90%	–	–	
					Hydrogel + BM + OP9 + factors	55.40%	-	-–	
					Hydrogel + BM cells + OP9DL1 + factors	78.30% (CD34 +)	The greatest CFU potential	Lymphoid, myeloid and erythroid	
Sugimine et al. (2016)	Human-ESCs and iPSCs	Collagen sponges (CSs) based-PET	6 days/2.5*_*104	CellGro-34	KhES1 + 2D culture	~ 7.5%	–	–	[219]
				bFGF/TPO/BMP4/FLt3/VEGF/SCF/	KhES1 + 3D culture	~ 10.5%	30 day colony-forming		
				IL-3/GM-CSF/M-CSF/EPO	201B7 + 3D culture	~ 3%	Activity and myeloid clones bias		
					402B2 + 3D culture	~ 6%	–		
					CB-A11 + 3D culture	~ 9%	–		
						(CD34 +)	–		
Shan et al. (2020)	Mouse-PSCs	Hydrogel	6–8 days/mPSCs -derived EBs	IMDM	EBs formation + 3D hydrogel	c-kit 5.2%	Myeloid and erythroid	Myeloid and lymphoid	[220]
				BMP4/Activin-A/VEGF/		CD41 32.7%	Potential differentiation	Cells (3 week)	
				FLt3L/SCF/TPO/IL-3/IL-6		CD45 18.4%			

*HA* hydroxyapatite, *PCL*, polycaprolactone, *PES* polyethersulfone, *PU* polyurethane, *PET* polyethylene terephthalate, *PLLA* Poly l-lactic acid, *TCP* tissue culture plastic, *ZTG* Zwitterionic poly-based hydrogels, *TNC* total nucleated cell, *TCC* total cell count, *TPO* thrombopoietin, *SCF* stem cell factor, *VEGF* vascular endothelial-derived growth factor, *GM-CSF* granulocyte–macrophage colony-stimulating factor, *M-CSF* macrophage colony-stimulating factor, *EPO* erythropoietin, *IL-3* Interleukin-3, *IL-6* Interleukin-6, *PGE2* prostaglandin E2, *LIF* leukemia inhibitory factor, *BMP4* bone morphogenetic protein 4, *FLt3(L)* fms-related tyrosine kinase 3 (ligand), *bFGF* basic fibroblast growth factor; EBs, embryonic bodies

Besides, the application of fluid shear stress mimicking cardiovascular forces within the AGM niche is another manageable means to attain optimal mechanical conditions, which can be readily included into scalable bioreactors. Flow experiments exposing cells (differentiating ESCs, embryonic HE or HSCs) to a defined pattern of laminar shear stress can be generated by a 2D adherent parallel plate configuration or through the assembly of a microfluidic platform [96, 99, 208]. Different mechanical parameters like varying modes (pulsatile or continuous) and magnitudes (1–10 dyn/cm^2^) of fluid shear stress can be investigated predictably and systematically, among which a shear stress of 5 dyn/cm^2^ is a value roughly amounting to the physiological magnitude experienced by cells in the dorsal aorta of E10.5 mouse embryos [86]. By seeding cells on a stretchable micropost array (SµPA) cytometry, Weng et al. employed varying degrees of defined static equibiaxial cell stretches [163]. Alginate beads with osteoblasts encapsulated were used to bio-mimic the low 3D stiffness of the BM niche [209]. The application of intermittent hydrostatic pressure (IHP) also is a widely used approach to simulate the BM mechanical microenvironment [69, 209, 210].

## Conclusion and perspective

The acquisition of sufficient functional HSCs in vitro prevails as a holy grail in the field of hematopoiesis that holds great promise for clinic-scale therapeutics. The identification of specific cues during HSC development and the fundamental grasp of mysteries in the underlying mechanism will be of help to decipher key points required for prolonged proliferation and pluripotency of HSCs. Significantly, the incorporation of biomechanical signals is a groundbreaking replenished theory that has revolutionized the scientific community’s current understanding of HSC regulatory signal types. This manuscript attempts to provide an integrated picture of the biophysical processes and probable mechanisms driving the phenotypic and functional outputs of HSCs. Besides, the practical aspects of mechanical signals are outlined with the focus on the mechanical mimicry platforms and the application of mechanical forces in an attempt to derive or expand HSCs in vitro.

Albeit certain mechanosensors and mechano-activated signaling pathways have been uncovered in several cell types and processes, some critical unanswered questions should be emphasized, such as, how natural fluctuations in the pattern, magnitude and duration of mechanical stimuli result in the discrepancy of genetic/epigenetic variations in HSCs, the precise definition of mechanical components presented in synthetic niches, and several pieces of evidence proved that biomechanical activation can successfully specify HSCs in vitro, but how long mechanical effects will last or whether they will be retained in transplant recipients, all of which remain to be explored and are interesting avenues for future research. In addition, aforementioned molecules or structures involved in cellular mechanotransduction and downstream events are highly interconnected rather than mutually exclusive. Several quintessential examples should be cited that, the primary cilium is actually a microtubular extension of cytoskeleton, whose basal body region is particularly a microtubule organizing center [221]. Cilium bending induces cytoskeletal deformation and membrane stretching at the base of the primary cilium [222], initiating extracellular Ca2 + influx through calcium channels in the ciliary membrane [223].

Cell junction receptors and associated proteins generally represent a mechanical linkage between the ECM and actin cytoskeleton. In the face of mechanical stimuli, adhesion complexes actively facilitate cytoskeleton assembly and stress fibers made of F-actin support the maturation and stabilization of focal adhesion. Therefore, the integrins-FAs-F-actin axis obviously works as an integrated whole in the process of mechanotransduction [46, 196, 197, 224–226]. In this respect, the regulation of biomechanical signals is a multifactorial event that can hardly be replicated by the manipulation of a single signaling axis, which may be why the interpretation of results obtained from synthetic niches seems to be challenging. Due to the complexity of these mechanical interactions, deeper mining is required to not only reveal the integrated characterization of various mechanical regulatory networks in a temporal and spatial manner at the cellular level, but also dissect the relative contribution of individual signaling in the related process.

In conclusion, we have summarized how biomechanical cues are pivotal for offering robust and instructive principles in HSC fate determination. Nevertheless, mechanical signals as regulators of HSC biology are still in their infancy, and the identification of several known mechanosensitive and mechanotransductive components suggests the possibility of more awaiting discovery. Fortunately, it is not naive to assume that researchers may be one step closer to fully appreciate the overall perspective of the mechanical regulatory paradigm within HSCs in the future because of the burgeoning experimental techniques ranging from high spatio-temporal resolution imaging to CRISPR/Cas9 gene-editing technique and bioengineering, single-cell sequencing and mass spectrometry. Mechanical inclusion strategy may be a favorable approach more suitable for HSC support with an expectation for the accelerated translation of mechanistic knowledge into the development of HSC-based regenerative medicine technologies.
